# Anticancer and Antiangiogenic Activities of Novel α-Mangostin Glycosides in Human Hepatocellular Carcinoma Cells via Downregulation of c-Met and HIF-1α

**DOI:** 10.3390/ijms21114043

**Published:** 2020-06-05

**Authors:** Sung Min Kim, Jang Mi Han, Tuoi Thi Le, Jae Kyung Sohng, Hye Jin Jung

**Affiliations:** 1 Department of Pharmaceutical Engineering and Biotechnology, Sun Moon University, Asan 31460, Korea; tjdals8855@gmail.com (S.M.K.); sohng@sunmoon.ac.kr (J.K.S.); 2 Department of Life Science and Biochemical Engineering, Sun Moon University, Asan 31460, Korea; gkswkdal200@naver.com (J.M.H.); tuoibioch@gmail.com (T.T.L.)

**Keywords:** hepatocellular carcinoma, α-mangostin glycosides, apoptosis, autophagy, tumor angiogenesis, c-Met, HIF-1α, cancer stemness

## Abstract

Hepatocellular carcinoma (HCC) is the most common type of primary liver cancer and is a leading cause of cancer-related death worldwide. Therefore, exploring effective anticancer agents and their modes of action is essential for the prevention and treatment of HCC. Glycosylation can significantly improve the physicochemical and biological properties of small molecules, such as high solubility, stability increase, and lower toxicity. In the present study, for the first time, we evaluated the anticancer and antiangiogenic activities of α-mangostin-3-*O*-*β*-D-2-deoxyglucopyranoside (Man-3DG) and α-mangostin 6-*O*-*β*-D-2-deoxyglucopyranoside (Man-6DG), glycosides of α-mangostin, against human HCC cells. Our results demonstrated that Man-3DG and Man-6DG significantly suppressed the growth of three different HCC cells (Hep3B, Huh7, and HepG2) as well as the migration of Hep3B cells. Furthermore, they induced cell cycle arrest in the G0/G1 phases and apoptotic cell death by regulating apoptosis-related proteins of mitochondria in Hep3B cells. Noticeably, Man-3DG and Man-6DG also caused autophagy, while co-treatment of the α-mangostin glycosides with an autophagy inhibitor 3-MA enhanced the inhibitory effect on Hep3B cell growth in comparison to single agent treatment. Moreover, Man-3DG and Man-6DG inhibited the c-Met signaling pathway that plays a critical role in the pathogenesis of HCC. Furthermore, the α-mangostin glycosides decreased Hep3B cell-induced angiogenesis in vitro through the downregulation of hypoxia-inducible factor-1α (HIF-1α) and vascular endothelial growth factor (VEGF). Notably, Man-6DG more effectively inhibited the growth, tumorsphere formation, and expression of cancer stemness regulators compared to α-mangostin and Man-3DG in 3D spheroid-cultured Hep3B cells. These findings suggest that the α-mangostin glycosides might be promising anticancer agents for HCC treatment with superior pharmacological properties than the parent molecule α-mangostin.

## 1. Introduction

Hepatocellular carcinoma (HCC) is one of the most prevalent cancers in the world and it accounts for 85%–90% of all cases of liver cancer. It has been reported as the second most prevalent cause of global cancer mortality [1,2]. HCC is a multifactorial disease that is caused by smoking, alcohol, mycotoxins, human hepatitis virus, fatigue, and obesity [3]. The current therapy for HCC includes surgical operation, radiofrequency ablation, and chemotherapy. However, the therapeutic outcomes are unsatisfactory due to metastasis and a high recurrence rate [4]. Therefore, it is still necessary to develop new anticancer drugs for the prevention and treatment of HCC.

Accumulating evidence has identified that c-Met was overexpressed in HCC tumors, and aberrant c-Met activity contributed to a poor prognosis [5,6,7]. The c-Met is a receptor tyrosine kinase that binds to hepatic growth factor (HGF). It regulates the activation of multiple cellular downstream signaling pathways including, mitogen activated protein kinase (MAPK), phosphatidylinositol-3 kinase (PI3K)/protein kinase B (AKT), signal transducer and activator of transcription (STAT), β-catenin, and Notch pathways [8,9]. The upregulation of these c-Met-mediated signaling pathways was implicated in the development and progression of HCC, suggesting that the inhibition of c-Met signaling may have a therapeutic potential for HCC.

HCC is one of the most hypoxic tumors that induces angiogenesis that is the formation of new blood vessels for the supply of nutrients and oxygen within the tumor [10]. Since the growth of HCC depends on angiogenesis, an antiangiogenic agent sorafenib has been approved for the advanced stage of HCC treatment [11]. Hypoxia-inducible factor-1 (HIF-1) is a well-defined critical mediator of cell response to hypoxia [12]. Under hypoxic condition, an oxygen-sensitive HIF-1α is induced that forms a heterodimer with a constitutively expressed HIF-1β and transactivates various kinds of hypoxia-inducible genes that are involved in energy/iron metabolism, angiogenesis, erythropoiesis, cell proliferation, and cell survival decisions. Recent studies have revealed that the overexpression of HIF-1α in HCC is closely associated with tumor angiogenesis, metastasis, treatment resistance, and poor prognosis [13,14]. Therefore, targeting HIF-1α can be a powerful strategy to control tumor angiogenesis in HCC.

α-Mangostin is a polyphenolic xanthone that is isolated from the pericarp of a tropical fruit mangosteen (*Garcinia mangostana* Linn.) [15,16]. The phytochemical has been reported to possess a variety of biological activities, such as antioxidant, anti-inflammatory, antitumor, antidiabetic, antibacterial, antifungal, anti-obesity, cardioprotective, and neuroprotective effects. Various in vitro and in vivo studies have demonstrated that α-mangostin has chemopreventive and chemotherapeutic potential against a wide range of cancer cell types. It shows antiproliferative, proapoptotic, antiangiogenic, and antimetastatic activities [17,18,19]. However, the low oral bioavailability of α-mangostin that is due to its first-pass metabolism, poor absorption due to low water solubility, and the efflux effect of P-glycoprotein, has limited its further clinical applications [20,21,22]. Therefore, designing novel α-mangostin analogs is required to improve its bioavailability.

The glycosylation of bioactive compounds has been considered as a promising strategy to improve their bioavailability by inducing changes in their physicochemical properties [23,24,25]. The conjugation of sugars to the compounds could facilitate biodistribution in tissues, penetration through biological membranes, metabolic stabilization, receptor-binding, the stability of labile molecules, the reduction of toxicity, the modification of biological activities, and water solubility. To overcome the poor physicochemical properties of α-mangostin, such as low water solubility, six novel glycoside derivatives of the natural compound have been recently produced through biocatalytic glycosylation reactions [26]. All the α-mangostin glycosides exhibited an improved water solubility, and several analogs among them showed an increased antibacterial activity against Gram-positive bacteria compared to α-mangostin. However, the anticancer property of the α-mangostin glycosides has not yet been investigated.

**Figure 1:**
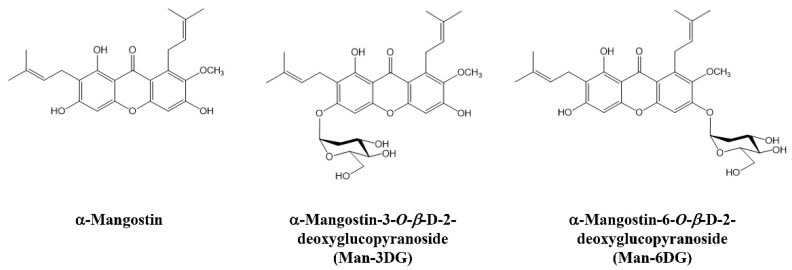
The chemical structures of α-mangostin and α-mangostin glycosides.

In our preliminary study, among six α-mangostin glycosides, α-mangostin 3-*O*-*β*-D-2-deoxyglucopyranoside (Man-3DG) and α-mangostin 6-*O*-*β*-D-2-deoxyglucopyranoside (Man-6DG) showed the most potent antiproliferative effect on several cancer cell lines, including HCC. In the present study, we intensively evaluated the anticancer activity of Man-3DG and Man-6DG against human hepatoma cells (Figure 1). The results demonstrated that Man-3DG and Man-6DG suppress the growth, migration, tumor angiogenesis, and cancer stemness of HCC through the inhibition of c-Met signaling and expression of HIF-1α and stemness markers. These findings suggest that the glycoside analogs of α-mangostin might be implicated as promising chemotherapeutic agents for HCC treatment with refined pharmacological property compared to the native compound.

## 2. Results

### 2.1. Effects of α-Mangostin Glycosides on the Growth of HCC Cells

**Figure 2:**
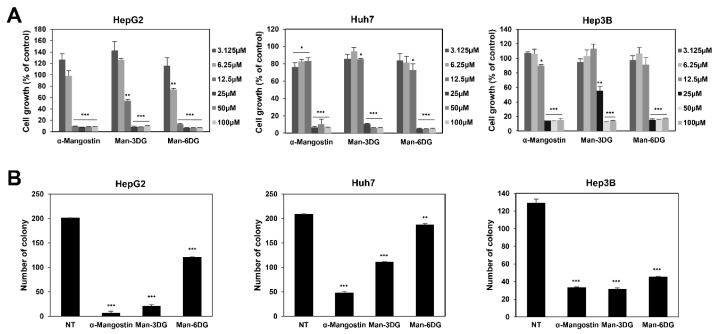
The growth-inhibitory effects of α-mangostin glycosides on hepatocellular carcinoma (HCC) cells. (**A**) The effects of α-mangostin, Man-3DG, and Man-6DG on the growth of HepG2, Huh7, and Hep3B cells. The cells were treated with increasing concentrations of three compounds (0–100 µM) for 72 h, and cell growth was measured through the 3-(4,5-dimethylthiazol-2-yl)-2,5-diphenyltetrazolium bromide (MTT) assay. Data are presented as percentage relative to dimethyl sulfoxide (DMSO)-treated control (% of control). Each value represents the mean ± SD from three independent experiments. (**B**) The effects of α-mangostin, Man-3DG, and Man-6DG on the colony forming ability of HepG2, Huh7, and Hep3B cells. The cells were incubated in the absence or presence of the three compounds (10 µM) for 7 days. The cell colonies were detected through crystal violet staining. Each value represents the mean ± SD from three independent experiments. * *p* < 0.05, ** *p* < 0.01, and *** *p* < 0.001 versus the control.

We first examined the effects of α-mangostin glycosides, Man-3DG, and Man-6DG, on the growth of three different HCC cell lines (HepG2, Huh7, and Hep3B). The cells were treated with α-mangostin, Man-3DG, and Man-6DG (0–100 μM) for 72 h and cell growth was then measured using the 3-(4,5-dimethylthiazol-2-yl)-2,5-diphenyltetrazolium bromide (MTT) assay. As shown in Figure 2A, the α-mangostin glycosides potently inhibited the growth of HepG2, Huh7, and Hep3B cells (IC_50_ = 7.3, 15.9, and 13.1 μM by α-mangostin, respectively; IC_50_ = 12.6, 22.3, and 25.0 μM by Man-3DG, respectively; IC_50_ = 7.0, 14.7, and 12.5 μM by Man-6DG, respectively). Particularly, Man-6DG showed a growth inhibitory effect on HCC cells to a similar extent as that of the α-mangostin.

Thereafter, we investigated the effect of Man-3DG and Man-6DG on the clonogenic growth of HCC cells. As shown in Figure 2B, the colony formation of HepG2, Huh7, and Hep3B cells was inhibited following the treatment with α-mangostin, Man-3DG, and Man-6DG using a concentration of 10 μM. However, Man-3DG and Man-6DG had a lower ability to suppress the colony formation of HCC cells compared to α-mangostin. Collectively, these results indicate that the glycoside analogs of α-mangostin did not exhibit a better growth inhibitory activity than α-mangostin. However, the α-mangostin glycoside analogs were observed to possess the antiproliferative effect against HCC cells. In subsequent studies, we focused on Hep3B cells, in which the α-mangostin glycosides showed the prominent growth inhibitory effect in both assays.

### 2.2. Effects of α-Mangostin Glycosides on the Migration of Hep3B Cells

**Figure 3:**
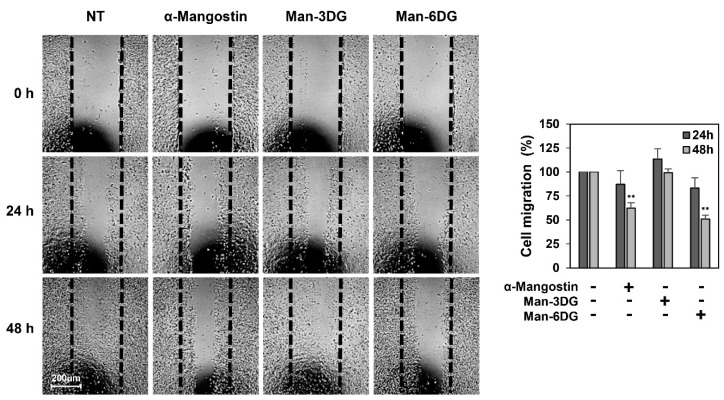
The effects of α-mangostin glycosides on the migration of Hep3B cells by a wound-healing assay. The cells were incubated in the absence or presence of α-mangostin, Man-3DG, and Man-6DG (10 µM) for 48 h. The cells that migrated into the gap were counted using an optical microscope. The dotted black lines indicate the edge of the gap at 0 h. Each value represents the mean ± SD from three independent experiments. ** *p* < 0.01 versus the control.

To evaluate the effects of Man-3DG and Man-6DG on the migration of HCC cells, a wound-healing assay was conducted using Hep3B cells. The results showed that Man-6DG, and not Man-3DG, significantly decreased the migration of Hep3B cells at 48 h after treatment compared to the control cells, as observed for α-mangostin (Figure 3). Therefore, Man-6DG may have the potential to inhibit the metastasis of HCC cells.

### 2.3. Effects of α-Mangostin Glycosides on the Apoptosis of Hep3B Cells

**Figure 4:**
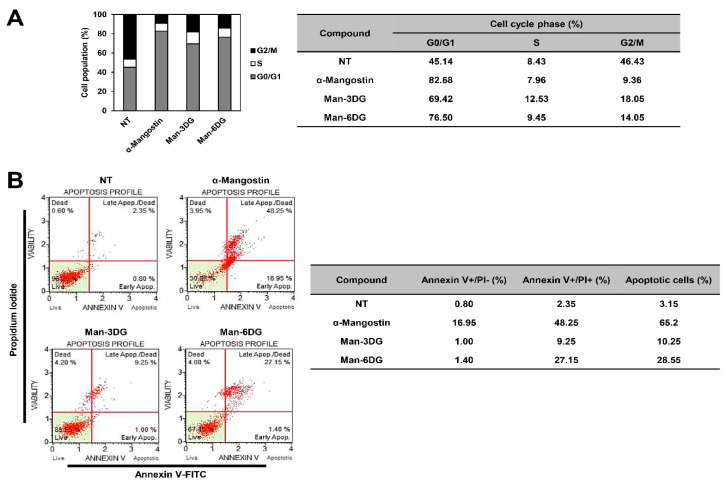
The effects of α-mangostin glycosides on the cell cycle and apoptotic cell death of Hep3B cells. (**A**) The cell cycle distribution of Hep3B cells was evaluated through flow cytometry after the treatment of α-mangostin, Man-3DG, and Man-6DG (10 µM) for 24 h. (**B**) Hep3B cells were treated using three compounds (40 µM) for 24 h. Apoptotic cells were determined through flow cytometry analysis following annexin V-FITC and propidium iodide (PI) dual labeling.

Abnormal cell cycle progression and the evasion of apoptosis are common features of cancer. Thus, induction of cell cycle arrest and apoptosis in cancer cells is considered as a key cellular mechanism of action of anticancer drugs [27,28]. To determine whether α-mangostin glycosides inhibit the HCC cell growth by causing arrest in a specific phase of cell cycle, we investigated the effects of Man-3DG and Man-6DG on the cell cycle distribution of Hep3B cells through flow cytometric analysis. As shown in Figure 4A, α-mangostin, Man-3DG, and Man-6DG caused a significant increase in cell population at the G0/G1 phase along with a remarkable decrease in cell population in the G2/M phase in comparison to the untreated control cells. These data demonstrate that α-mangostin and its glycosides suppressed the growth of Hep3B cells through the cell cycle arrest at G0/G1 phase.

We further explored whether the antiproliferative effects of Man-3DG and Man-6DG against HCC cells were related to apoptotic cell death through flow cytometric analysis after the Annexin V-FITC/PI double staining. Our results showed that treatment with α-mangostin, Man-3DG, and Man-6DG significantly enhanced the apoptosis of Hep3B cells, with an apoptotic rate of 3.15% in untreated control, 65.2% in α-mangostin, 10.25% in Man-3DG, and 28.55% in Man-6DG (Figure 4B). Moreover, Man-6DG showed a more potent apoptosis-inducting effect compared to Man-3DG.

**Figure 5:**
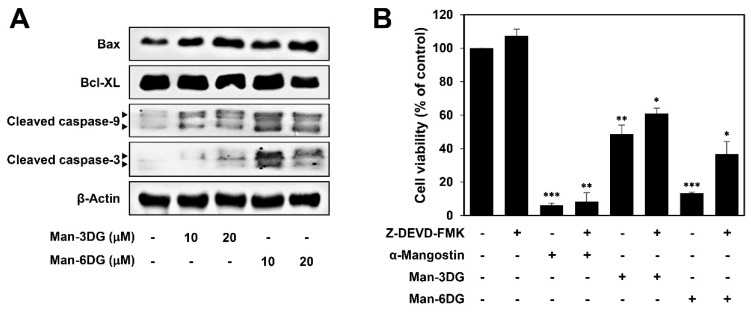
The effects of α-mangostin glycosides on the expression of mitochondrial-mediated apoptosis regulators in Hep3B cells. (**A**) The cells were treated with Man-3DG and Man-6DG (10 and 20 µM) for 24 h, and the protein levels were detected through western blot analysis using specific antibodies. The levels of β-actin were used as an internal control. (**B**) The cells were treated with α-mangostin, Man-3DG, and Man-6DG (10 μM) in the presence or absence of Z-DEVD-FMK (20 μM) for 24 h. The cell viability was measured through MTT assay. Each value represents the mean ± SD from three independent experiments. * *p* < 0.05, ** *p* < 0.01, and *** *p* < 0.001 versus the control.

It has been reported that α-mangostin induces mitochondrial dependent apoptosis in HCC cells [29]. We, therefore, assessed the effects of Man-3DG and Man-6DG on the expression of the critical regulators of mitochondrial-mediated apoptosis in Hep3B cells. As shown in Figure 5A, Man-3DG and Man-6DG increased the protein levels of cleaved caspase-3, cleaved caspase-9, and Bax, whereas the protein level of Bcl-XL was decreased. Particularly, Man-6DG more effectively regulated the expression of these apoptosis-related proteins than Man-3DG in accordance with a stronger apoptosis-inducing effect of Man-6DG. Moreover, a caspase-3 inhibitor Z-DEVD-FMK partially rescued the viability of Hep3B cells that was inhibited by the α-mangostin glycosides (Figure 5B). Collectively, these results suggest that the cytostatic and cytotoxic effects by the induction of G0/G1 cell cycle arrest and mitochondrial caspase-dependent apoptosis may contribute to the anticancer activities of Man-3DG and Man-6DG on HCC cells.

### 2.4. Effects of α-Mangostin Glycosides on the Autophagy of Hep3B Cells

**Figure 6:**
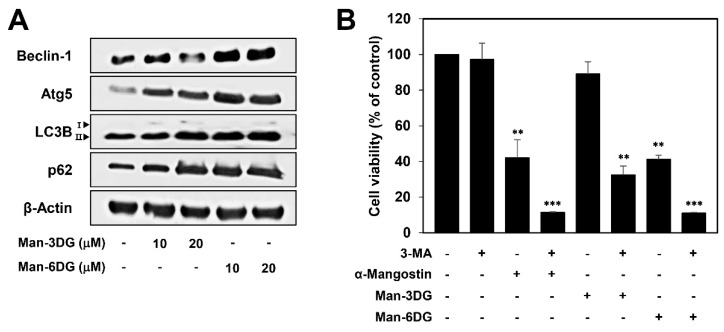
The effects of α-mangostin glycosides on the autophagy of Hep3B cells. (**A**) The effects of Man-3DG and Man-6DG on the expression of autophagy-related proteins in Hep3B cells. The cells were treated with Man-3DG and Man-6DG (10 and 20 μM) for 24 h, and the protein levels were detected through western blot analysis using specific antibodies. The levels of β-actin were used as an internal control. (**B**) The effects of α-mangostin, Man-3DG, and Man-6DG on Hep3B cell viability in combination with an autophagy inhibitor. Hep3B cells were treated with the three compounds (20 μM) in the presence or absence of 3-MA (1 mM) for 24 h. The cell viability was measured through MTT assay. Each value represents the mean ± SD from three independent experiments. ** *p* < 0.01 and *** *p* < 0.001 versus the control.

Autophagy is a cellular catabolic pathway that is involved in lysosomal degradation and the recycling of proteins and organelles [30]. Accumulating evidence has revealed that autophagy participates in both cell death and cell survival [31,32]. In a recent study, α-mangostin-induced autophagy was seen to protect human breast cancer cells from apoptotic death [33]. To confirm whether the activation of autophagy is also related to the anticancer effects of α-mangostin glycosides in HCC cells, we investigated the regulatory effects of Man-3DG and Man-6DG on the expression of autophagy-related proteins in Hep3B cells through western blotting. As shown in Figure 6A, the protein levels of Atg5, Beclin-1, LC3B-II, and p62 were upregulated in Hep3B cells after treatment with the α-mangostin glycosides, suggesting that Man-3DG and Man-6DG can trigger autophagy activation as well as apoptotic cell death in HCC cells.

To further understand the role of autophagy and apoptosis induced by α-mangostin glycosides in HCC cells, Hep3B cells were treated with α-mangostin, Man-3DG, and Man-6DG in the presence or absence of 3-MA, an autophagy inhibitor. As shown in Figure 6B, combined treatment of α-mangostin and its glycosides with 3-MA more potently decreased the viability of Hep3B cells in comparison to the treatment with α-mangostin, Man-3DG, and Man-6DG alone. These results imply that the activation of autophagy induced by α-mangostin, Man-3DG, and Man-6DG may play a protective role in the apoptotic cell death that they caused in HCC cells. Therefore, co-treatment with autophagy inhibitor can be a promising strategy for improving the anticancer effects of α-mangostin glycosides against HCC.

### 2.5. Effects of α-Mangostin Glycosides on the c-Met Signaling Pathway in Hep3B Cells

**Figure 7:**
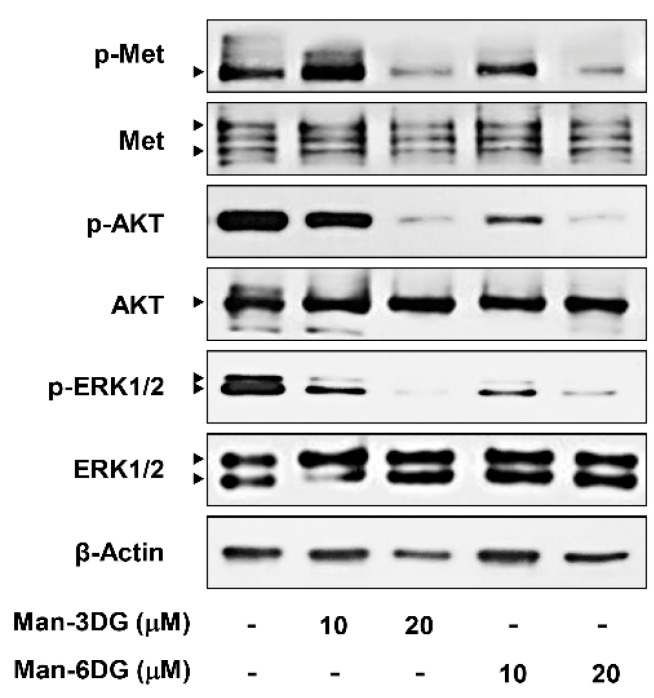
The effects of α-mangostin glycosides on c-Met signaling pathway in Hep3B cells. The cells were treated with Man-3DG and Man-6DG (10 and 20 µM) for 24 h, and the protein levels were detected through western blot analysis using specific antibodies. The levels of β-actin were used as an internal control.

Aberrant c-Met signaling is one of the crucial drivers of HCC progression. Several downstream signaling pathways, including MAPK and PI3K/AKT, can be activated by c-Met, and thereby upregulate the growth and metastasis of HCC cells [7,8,9]. We, therefore, determined whether α-mangostin glycosides affect the activation of c-Met and its downstream effectors, p44/42 MAPK (ERK1/2) and AKT, in Hep3B cells. As shown in Figure 7, Man-3DG and Man-6DG efficiently suppressed the phosphorylation of c-Met, AKT, and ERK1/2 without inhibiting the total protein levels, suggesting that the α-mangostin glycosides may exhibit the anticancer activities against HCC cells through the downregulation of c-Met-mediated signaling cascades.

### 2.6. Effects of α-Mangostin Glycosides on the Hep3B Cell-Induced Angiogenesis

**Figure 8:**
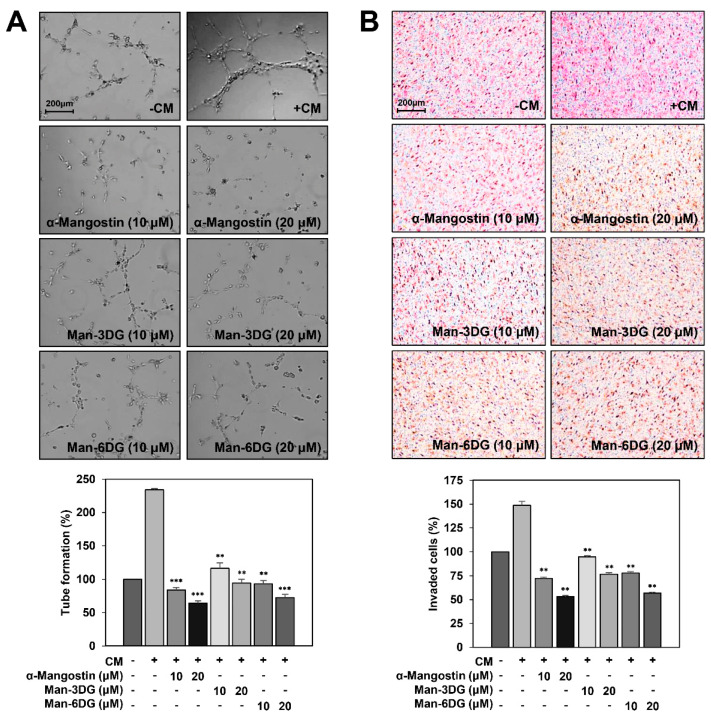
The effects of α-mangostin glycosides on the Hep3B cell-induced angiogenesis. The tumor cell-induced angiogenesis was assessed using (**A**) a conditioned medium from tumor cells for in vitro tube formation assay, and (**B**) an in vitro co-culture system based on the chemoinvasion assay. Hep3B cells were treated with 10 and 20 μM of α-mangostin, Man-3DG, and Man-6DG for 24 h. (**A**) The basal level of the tube formation of human umbilical vein endothelial cells (HUVECs) that were treated with non-conditioned medium without Hep3B cells was normalized to 100%. Each value represents the mean ± SD from three independent experiments. ** *p* < 0.01 and *** *p* < 0.001 versus the conditioned medium from untreated Hep3B cells. (**B**) The basal level of the invasiveness of HUVECs that were incubated in a serum-free medium without Hep3B cells was normalized to 100%. Each value represents the mean ± SD from three independent experiments. ** *p* < 0.01 versus the control with untreated Hep3B cells.

As angiogenesis is essential for the growth and metastasis of HCC cells, the inhibition of tumor cell-induced angiogenesis is a powerful strategy to restrict HCC progression [10,11]. In addition, it has been reported that α-mangostin has an antiangiogenic activity [34]. To evaluate whether α-mangostin glycosides suppress HCC cell-induced angiogenesis, the effects of α-mangostin, Man-3DG, and Man-6DG on the tube formation and invasion of human umbilical vein endothelial cells (HUVECs) stimulated by Hep3B cells were examined. As shown in Figure 8A, the conditioned medium (CM) from Hep3B cells induced the tube formation of HUVECs as compared to the control medium. However, the CM treated with α-mangostin, Man-3DG, and Man-6DG remarkably blocked the tube formation of HUVECs.

We further investigated the effects of α-mangostin, Man-3DG, and Man-6DG on the invasion of HUVECs induced by Hep3B cells using a co-culture assay. As a result, the HUVECs co-cultured with Hep3B cells showed significant invasion compared to HUVECs (Figure 8B). However, Hep3B cells treated with α-mangostin, Man-3DG, and Man-6DG effectively prevented the increased invasion of HUVECs, implying that the α-mangostin glycosides can inhibit HCC cell-induced angiogenesis.

### 2.7. Effects of α-Mangostin Glycosides on the Hypoxia-Induced Accumulation of HIF-1α Protein

**Figure 9:**
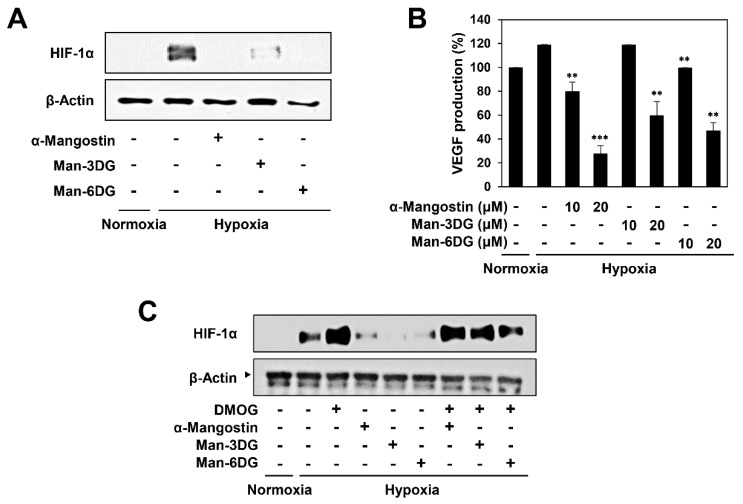
The effects of α-mangostin glycosides on HIF-1α activity in Hep3B cells. (**A**) The effects of α-mangostin, Man-3DG, and Man-6DG on the HIF-1α protein accumulation induced by hypoxia. Hep3B cells were pretreated with the three compounds (10 μM) for 1 h and then exposed to 1% O_2_ for 4 h. The protein levels of HIF-1α were detected through western blot analysis. The levels of β-actin were used as an internal control. (**B**) The effects of α-mangostin, Man-3DG, and Man-6DG on VEGF expression. Hep3B cells were pretreated with the three compounds (10 and 20 μM) for 1 h and then exposed to 1% O_2_ for 6 h. The concentration of vascular endothelial growth factor (VEGF) protein in the culture supernatant was determined through a VEGF specific ELISA. Each value represents the mean ± SD from three independent experiments. ** *p* < 0.01 and *** *p* < 0.001 versus the hypoxic control. (**C**) The effects of α-mangostin, Man-3DG, and Man-6DG on the HIF-1α protein accumulation in the presence or absence of dimethyloxalylglycine (DMOG). Hep3B cells were pretreated with the three compounds (10 μM) and DMOG (1 mM) for 1 h and then exposed to 1% O_2_ for 4 h. The protein levels of HIF-1α were detected through western blot analysis. The levels of β-actin were used as an internal control.

HIF-1α is known as the most potent inducer of tumor angiogenesis that regulates the expression of proangiogenic factors, such as vascular endothelial growth factor (VEGF) [12,13,14]. To assess the contribution of HIF-1α in mediating the tumor angiogenesis inhibitory effect of α-mangostin glycosides, the effects of α-mangostin, Man-3DG, and Man-6DG on the HIF-1α expression of Hep3B cells were determined through western blotting. As revealed in Figure 9A, all of them markedly inhibited the hypoxia-induced accumulation of HIF-1α protein in Hep3B cells.

Furthermore, we confirmed the effects of α-mangostin glycosides on the expression of VEGF through ELISA. The VEGF production of Hep3B cells under hypoxic condition was prominently reduced by treatment with α-mangostin, Man-3DG, and Man-6DG (Figure 9B). These data indicate that the α-mangostin glycosides might inhibit HCC cell-induced angiogenesis by suppressing the expression of HIF-1α and its downstream target VEGF.

In addition, we assessed whether the HIF-1α inhibitory effect by α-mangostin glycosides was associated with prolyl-hydroxylase (PHD) activity. As shown in Figure 9C, treatment with a PHD inhibitor dimethyloxalylglycine (DMOG) suppressed the degradation of HIF-1α protein by α-mangostin, Man-3DG, and Man-6DG, suggesting that the compounds may decrease the protein stability of HIF-1α through the upregulation of PHD activity.

### 2.8. Effects of α-Mangostin Glycosides on the Tumorsphere Formation and Cancer Stemness of Hep3B Cells

**Figure 10:**
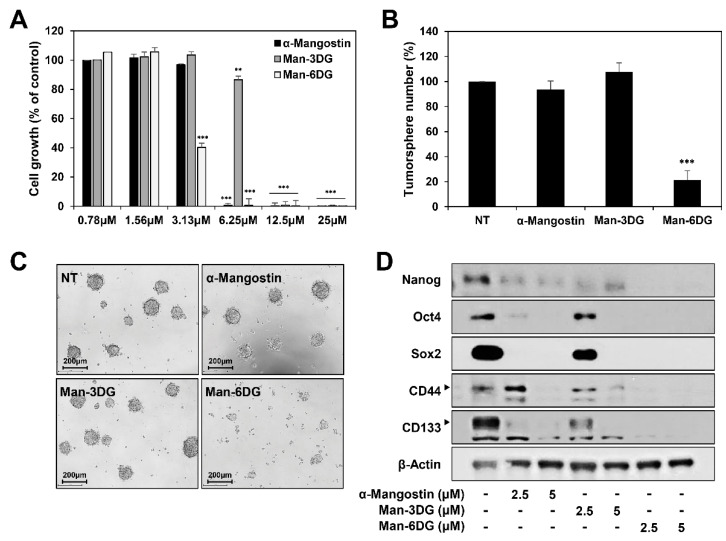
The effects of α-mangostin glycosides on the tumorsphere formation and cancer stemness of Hep3B cells. (**A**) The effects of α-mangostin, Man-3DG, and Man-6DG on the growth of 3D-cultured Hep3B cells. The cells were treated with increasing concentrations of three compounds (0–25 μM) in serum-free suspended spheroid culture condition for 7 days. Cell growth was measured by CellTiter-Glo^®^ luminescent cell viability assay. (**B**,**C**) The effects of α-mangostin, Man-3DG, and Man-6DG on the tumorsphere formation of Hep3B cells. The cells were treated with three compounds (3.13 μM) in the 3D culture environment for 7 days. The number of formed tumorspheres in each well was counted under an optical microscope. (**D**) The effects of α-mangostin, Man-3DG, and Man-6DG on the expression of cancer stemness markers in Hep3B cancer stem-like cells. The cells were treated with three compounds (2.5 and 5 µM) for 24 h, and the protein levels were detected by western blot analysis using specific antibodies. The levels of β-actin were used as an internal control. Each value represents the mean ± SD from three independent experiments. ** *p* < 0.01 and *** *p* < 0.001 versus the control.

A growing body of evidence suggests that that cell responses in three-dimensional (3D) culture are more similar to in vivo behavior compared to two-dimensional (2D) culture [35]. To further confirm the anticancer effect of α-mangostin glycosides in the 3D culture environment, Hep3B cells were grown in serum-free suspended spheroid culture condition [36]. As shown in Figure 10A, α-mangostin, Man-3DG, and Man-6DG potently inhibited the growth of 3D-cultured Hep3B cells (IC_50_ = 4.17, 9.99, and 2.98 μM, respectively). Notably, Man-6DG showed a better growth inhibitory effect on 3D-cultured HCC cells compared to α-mangostin. In addition, the tumorsphere forming ability of Hep3B cells was remarkably suppressed by treatment with 3.13 μM of Man-6DG, but not by α-mangostin and Man-3DG (Figure 10B,C). Man-6DG effectively reduced both the size and number of tumorspheres, in comparison with α-mangostin and Man-3DG at the indicated dose.

Liver cancer stem cells (CSCs) are currently considered as a specific subpopulation with significant tumorigenic potential, which should contribute to the development and recurrence of HCC [37]. CSCs can be isolated from cancer cell lines and grown in the 3D tumorsphere suspension cultures [38]. We thus investigated whether α-mangostin glycosides regulate the expression of the key stemness-related markers for HCC CSCs in the 3D tumorsphere culture condition of Hep3B cells. Treatment with α-mangostin, Man-3DG, and Man-6DG markedly decreased the expression levels of cancer stemness regulators, including Nanog, Oct4, Sox2, CD44, and CD133, in the 3D tumorsphere-cultured Hep3B cells (Figure 10D). Particularly, the suppressive effect of Man-6DG on the expression of cancer stemness markers was more potent than those of α-mangostin and Man-3DG. These data suggest that Man-6DG may possess a better bioavailability than α-mangostin in the in vivo environment as well as the superior therapeutic potential to eliminate HCC CSCs.

## 3. Discussion

To date, numerous chemotherapeutic agents used in cancer therapy have been derived from natural products [39]. However, many promising natural drug candidates encounter diverse problems, such as off-target cytotoxicity, poor water solubility, limited permeability, and low bioactivity [40,41]. Therefore, researchers are constantly looking for ways that can help to lower the cytotoxicity and increase bioavailability of these drugs. It has been reported that α-mangostin, a natural xanthone, has potent anticancer effects against various types of cancer [17,18,19]. However, the poor water solubility and low bioavailability of α-mangostin led to various limitations in clinical applications [20,21,22]. Several attempts, including the modification of its chemical structure and employment of a dispersion matrix, have been made to improve the pharmacological properties of α-mangostin [42,43]. Recently, six novel α-mangostin glycosides (three glucopyranosides and three 2-deoxyglucopyranosides of α-mangostin) have been produced through the application of biocatalytic glycosylation reactions using a *Bacillus* glycosyltransferase (YjiC) and sugar donors, including UDP-α-D-glucose and UDP-α-D-2-deoxyglucose [26]. As observed for curcumin glycosides with enhanced anticancer activity compared to curcumin, the glycosylation of bioactive compounds can change their physicochemical properties, and thereby improve their bioavailability at the cell or organism level [23,24,25,44]. Indeed, the α-mangostin glycosides showed better water solubility than the parent compound. Interestingly, the glucopyranosides of α-mangostin exhibited superior antibacterial activity against Gram-positive bacteria compared to α-mangostin, whereas the 2-deoxyglucopyranoside derivatives did not [26]. However, antitumor activities of the α-mangostin glycosides have not been investigated yet.

**Figure 11:**
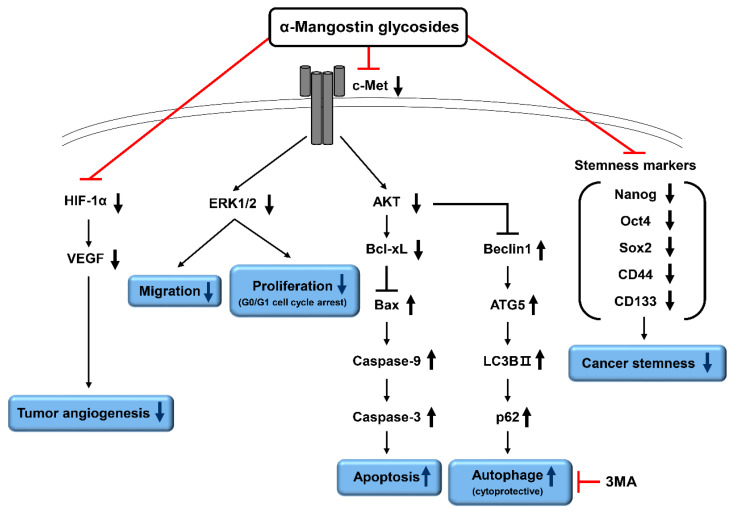
Proposed molecular mechanisms of anticancer action of α-mangostin glycosides in HCC cells. The α-mangostin glycosides, Man-3DG and Man-6DG, may exhibit potent anticancer and antiangiogenic activities against HCC cells by downregulating c-Met, HIF-1α, and cancer stemness regulators. In addition, the combination with autophagy inhibitor can be a promising strategy for improving the anticancer effects of α-mangostin glycosides against HCC.

In the present study, for the first time, we identified the anticancer effects and molecular mechanisms of action of the two α-mangostin glycosides, including α-mangostin 3-*O*-*β*-D-2-deoxyglucopyranoside (Man-3DG) and α-mangostin 6-*O*-*β*-D-2-deoxyglucopyranoside (Man-6DG), which showed most potent antiproliferative effect against HCC cells among six α-mangostin glycosides in our preliminary study. Our results showed that Man-3DG and Man-6DG effectively suppress the growth, migration, tumor angiogenesis, and cancer stemness of HCC cells in vitro (Figure 11).

Apoptosis and autophagy are two major types of programmed cell deaths, designated as type I and type II, respectively [30]. Apoptosis is characterized by the activation of caspase proteases, whereas autophagic cell death consists of complex processes to degrade intracellular components through autophagosomes and lysosomes. A wide range of studies have revealed that the activation of apoptosis signaling pathways is a key molecular mechanism of action of anticancer drugs [27]. The mitochondria-mediated caspase activation pathway is a major apoptotic pathway [45]. In response to various cellular stresses, proapoptotic members of the Bcl-2 family, including Bax, induce the mitochondrial outer membrane permeabilization, and subsequently, cytochrome c is released into the cytosol. In the cytosol, cytochrome c binds to apoptotic protease activating factor 1 (APAF1), and the complex leads to the activation of caspase-9. However, the release of cytochrome c can be inhibited by the antiapoptotic Bcl-2 proteins, such as Bcl-XL. Active caspase-9 cleaves and activates a key executioner enzyme, caspases-3. In a previous study, α-mangostin caused cell cycle arrest in the sub-G1 phase and mitochondrial dependent apoptosis in human hepatoma SK-Hep-1 cells [29]. In the present study, we have found that α-mangostin glycosides, Man-3DG and Man-6DG, as well as α-mangostin significantly induced cell cycle arrest in the G0/G1 phases and apoptosis in Hep3B cells. Furthermore, Man-3DG and Man-6DG upregulated the expression levels of Bax, cleaved caspase-9, and cleaved caspase-3, following the downregulation of Bcl-XL protein. These results suggest that the inhibition of HCC cell growth by the α-mangostin glycosides is involved in the induction of mitochondrial caspase-dependent apoptosis.

Accumulating evidence has revealed that natural anticancer compounds could regulate autophagy in human cancer cells [31,32]. Interestingly, it was shown that autophagy plays either survival/protective or nonapoptotic cell death-inducing role in cancer cells treated with natural compounds. Recent studies have reported that α-mangostin induced not only apoptosis, but also autophagy in chronic myeloid leukemia and breast cancer cells [33,46]. The provocation of autophagy resulted in a reduction in the apoptotic effect of α-mangostin. We, therefore, determined whether autophagy affects the anticancer effects of α-mangostin glycosides in HCC cells. In the autophagic signaling pathway, the initiation complex, including Beclin-1, is activated by AMP-activated protein kinase (AMPK) activation and/or mammalian target of rapamycin complex 1 (mTORC1) inhibition [47]. The Atg5-12 conjugate forms a complex to stabilize the phagophore, and finally generates a LC3-II conjugate. Because autophagosomes do not exhibit selectivity, the autophagy receptor p62 is necessary to link autophagosomes to specific proteins and binds directly to LC3 to degrade ubiquitinated protein through autophagy. In the present study, Man-3DG and Man-6DG increased the expression of autophagy-related proteins, such as Atg5, Beclin-1, LC3B-II, and p62. However, the inhibition of autophagy by 3-MA significantly enhanced the suppressive effects of α-mangostin, Man-3DG, and Man-6DG on the viability of HCC cells. Therefore, targeting the autophagy pathway may be a potential therapeutic strategy to enhance the apoptosis-inducing effects of α-mangostin glycosides against HCC cells.

The receptor tyrosine kinase c-Met that is encoded by hepatocyte growth factor receptor (HGFR) gene, has emerged as a potential therapeutic target in various tumors, including HCC [5,6,7]. The aberrant c-Met activity by mutation, amplification, or overexpression could promote tumor cell survival, growth, invasion, and metastasis through excessive stimulation of multiple downstream signaling pathways, including RAS/MAPK and PI3K/AKT pathways. Overexpressed c-Met has been observed in approximately 50% of HCC patients and associated with poor prognosis and short survival. Furthermore, inhibitors of c-Met signaling have shown antitumor effects in preclinical models of c-Met-positive HCC through the reduction of tumor cell proliferation, migration, and invasion and induction of apoptosis [8,9]. Therefore, blocking c-Met signaling can elicit more effective treatment of HCC. The results of the present study showed that Man-3DG and Man-6DG dramatically inhibited the activation of c-Met and its major downstream signaling effectors, including AKT and ERK1/2. This suggests that the α-mangostin glycosides may act as promising anticancer agents for treatment of c-Met-positive HCC.

The development of HCC that is one of the most hypoxic tumors essentially requires angiogenesis [10]. Therefore, the inhibition of tumor angiogenesis is considered a powerful strategy for HCC treatment [11]. The HIF-1 is a heterodimeric transcription factor that is composed of α and β subunits. It activates the transcription of a variety of genes that regulate cellular metabolism, survival, and angiogenesis in hypoxic conditions [12]. The activity of HIF-1 is regulated post-translationally by the oxygen-dependent HIF-1α subunit. In a normal oxygen environment, HIF-1α is hydroxylated by prolyl-hydroxylase (PHD), and rapidly degraded through the ubiquitin-proteasome pathway. However, during hypoxia, the activity of PHD is inhibited. Subsequently, the stabilized HIF-1α translocates into the nucleus and forms heterodimer with HIF-1β to bind with the HIF-responsive elements in promoters for activation of the transcription of downstream genes. Notably, the upregulation of HIF-1 activity promotes the expression of VEGF that is a critical inducer of tumor angiogenesis. Targeting HIF-1α, therefore, can have a therapeutic potential in HCC through the interruption of tumor angiogenesis [13,14]. Our data showed that α-mangostin, Man-3DG, and Man-6DG potently suppressed the in vitro angiogenic phenotypes stimulated by HCC cells. Furthermore, they significantly inhibited the hypoxia-induced HIF-1α accumulation and VEGF production in HCC cells, suggesting that the α-mangostin glycosides may inhibit tumor angiogenesis of HCC through the downregulation of HIF-1α and its target gene, VEGF. 

Three-dimensional spheroid cell culture is known to better stimulate in vivo cellular conditions in comparison with 2D cell culture and is widely used to increase a subpopulation of cancer cells with stem-cell-like properties, thereby providing new insights into cancer treatment and CSC research [35,38]. Our data revealed that Man-6DG more effectively inhibited the growth, tumorsphere formation, and cancer stemness regulators expression of Hep3B cells than α-mangostin and Man-3DG under a serum-free suspended spheroid culture condition. These results suggest that Man-6DG may possess a better bioavailability than α-mangostin in the in vivo environment as well as the superior therapeutic potential to eliminate HCC CSCs.

However, further investigations identifying the relationship between their structures and activities will be required to understand the differences in the anticancer effects between α-mangostin, Man-3DG, and Man-6DG. In previous study, the glucopyranosides of α-mangostin exhibited superior antibacterial activity to the 2-deoxyglucopyranoside derivatives [26]. On the other hand, our preliminary screening on the anticancer activity of six novel α-mangostin glycosides revealed that α-mangostin-2-deoxyglucopyranosides showed better growth-inhibitory effects on HCC cells than α-mangostin-glucopyranosides. In addition, in the present study, Man-6DG exhibited greater anticancer and antiangiogenic activities than Man-3DG in HCC cells. These findings imply that the conjugation of different glycosyl units to α-mangostin at different positions can change the conformational structures and physicochemical properties of the α-mangostin glycosides, resulting in the differences in the bioavailability and bioactivity between the glycosylated α-mangostin derivatives.

In summary, our study has identified the therapeutic potential of the α-mangostin glycosides, Man-3DG and Man-6DG, for HCC treatment. Although the improved water solubility of the glycosides did not cause superior anticancer and antiangiogenic activities to α-mangostin against HCC cells in vitro, the analogs effectively suppressed the growth, migration, and tumor angiogenesis of HCC cells through the blockade of c-Met signaling and HIF-1α expression. Furthermore, Man-6DG more effectively inhibited the growth, tumorsphere formation, and expression of cancer stemness regulators compared to α-mangostin and Man-3DG in 3D-cultured HCC cells. To clarify whether the α-mangostin glycosides can show the potent chemotherapeutic effects against HCC by increasing the bioavailability of α-mangostin, a further in vivo study using HCC preclinical models is required.

## 4. Materials and Methods

### 4.1. Materials

The α-mangostin, dimethyl sulfoxide (DMSO), 3-methyladenine (3-MA), dimethyloxalylglycine (DMOG), Z-DEVD-FMK, crystal violet, gelatin, and 3-(4,5-dimethylthiazol-2-yl)-2,5-diphenyltetrazolium bromide (MTT) were purchased from Sigma-Aldrich (St. Louis, MO, USA). The CellTiter-Glo^®^ luminescent cell viability assay kit was purchased from Promega (Madison, WI, USA). The α-mangostin glycosides, α-mangostin-3-*O*-*β*-D-2-deoxyglucopyranoside (Man-3DG) and α-mangostin 6-*O-β*-D-2-deoxyglucopyranoside (Man-6DG), were obtained through the application of biocatalytic glycosylation reactions [26]. The α-mangostin, Man-3DG, and Man-6DG were prepared at a concentration of 50 mM using DMSO. A fetal bovine serum (FBS), Dulbecco’s modified Eagle’s medium (DMEM), and minimum essential medium (MEM) were purchased from Gibco (Grand Island, NY, USA), and an endothelial growth medium-2 (EGM-2) and penicillin-streptomycin-amphotericin B were obtained from Lonza (Walkersville, MD, USA). Matrigel and Transwell chamber systems were obtained from BD Biosciences (San Jose, CA, USA) and Corning Costar (Acton, MA, USA), respectively. Anti-cleaved caspase-3, anti-cleaved caspase-9, anti-Bcl-XL, anti-Bax, anti-Atg5, anti-Beclin-1, anti-LC3B, anti-p62, anti-phospho-Met, anti-Met, anti-phospho-AKT, anti-AKT, anti-phospho-ERK1/2, anti-ERK1/2, anti-HIF-1α, anti-Nanog, anti-Oct4, anti-Sox2, anti-CD44, anti-CD133, and anti-β-actin antibodies were purchased from Cell Signaling Technology (Danvers, MA, USA).

### 4.2. Cell Culture and Hypoxic Conditions

The human hepatocellular carcinoma cells (HepG2, Huh7, and Hep3B) and human umbilical vein endothelial cells (HUVECs) were obtained from the Korean Cell Line Bank (Seoul, Korea) and the American Type Culture Collection (Manassas, VA, USA), respectively. The HepG2 and Huh7 cells were cultured in DMEM supplemented with 10% FBS and 1% antibiotics. The Hep3B cells and HUVECs were grown in MEM and EGM-2 supplemented with 10% FBS and 1% antibiotics, respectively. The cells were maintained at 37 °C in a humidified 5% CO_2_ incubator (Thermo Fisher Scientific, Vantaa, Finland). For hypoxic conditions, Hep3B cells were incubated in a hypoxic chamber (Forma Scientific, Marietta, OH, USA) under 5% CO_2_ and 1% O_2_ balanced with N_2_.

### 4.3. Cell Growth Assay

The HepG2, Huh7, and Hep3B cells (2 × 10^3^ cells/well) were seeded in 96-well culture plates. The cells were treated with various concentrations (0–100 μM) of α-mangostin, Man-3DG, and Man-6DG and incubated for 72 h. Thereafter, 50 μL of MTT solution (2 mg/mL) was added to each well and incubated for 3 h. After incubation, formazan crystals were dissolved in 100 μL of DMSO for each well. The absorbance of each well was determined at a wavelength of 540 nm using a microplate reader (Thermo Fisher Scientific).

### 4.4. Colony Formation Assay

The HepG2, Huh7, and Hep3B cells (5 × 10^2^ cells/well) were seeded in six-well culture plates and treated with 10 μM of α-mangostin, Man-3DG, and Man-6DG. After seven days, the cells were stained with crystal violet, and the number of colonies were counted for each well.

### 4.5. Wound Healing Assay

The Hep3B cells (3 × 10^5^ cells/well) were seeded in 24-well culture plates. The confluent monolayer cells were scratched using a tip, and each well was washed with phosphate buffered saline (PBS) to remove the floating cells. The cells were treated with 10 μM of α-mangostin, Man-3DG, and Man-6DG and then incubated for 48 h. The cell images were captured at different time-points (0, 24, and 48 h) under an optical microscope (Olympus, Center Valley, PA, USA) at a magnification of 100×.

### 4.6. Cell Cycle Analysis

The Hep3B cells (3 × 10^5^ cells/well) treated with 10 μM of α-mangostin, Man-3DG, and Man-6DG were trypsinized, washed with cold PBS, fixed with ice-cold 70% ethanol, and then stored overnight at −20 °C. The cells were washed with PBS and incubated in 100 μg/mL propidium iodide (PI) solution (Muse Cell Cycle Assay Reagent; Millipore, Hayward, CA, USA) for 30 min at room temperature in the dark. Cellular DNA content was analyzed using a flow cytometry (Muse Cell Analyzer; Millipore).

### 4.7. Apoptosis Analysis

The Hep3B cells (3 × 10^5^ cells/well) treated with 40 μM of α-mangostin, Man-3DG, and Man-6DG were harvested, washed with PBS, and then suspended using 100 μL of medium after staining with 100 μL of Annexin V-FITC and PI solution (Muse Annexin V & Dead Cell Reagent; Millipore) for 20 min at room temperature. The stained cells were analyzed by a flow cytometry (Muse Cell Analyzer; Millipore).

### 4.8. Western Blot Analysis

Following treatment with α-mangostin, Man-3DG, and Man-6DG (10 and 20 μΜ) for 24 h, the Hep3B cells were harvested and lysed in 2× Laemmli sample buffer (Bio-Rad Laboratories, Inc., Hercules, CA, USA). Cell lysates were subjected to 10% sodium dodecyl sulfate-polyacrylamide gel electrophoresis (SDS-PAGE), and the separated proteins were transferred to polyvinylidene difluoride (PVDF) membranes (Millipore) using standard electroblotting procedures. The blots were blocked using 5% or 1% skim milk at room temperature for 1 h and immunolabeled with primary antibodies against cleaved caspase-3, cleaved caspase-9, Bax, Bcl-XL, Atg5, Beclin-1, LC3B, p62, phospho-Met (Tyr1234/1235), Met, phospho-AKT (Ser473), AKT, phospho-ERK1/2 (Thr202/Tyr204), ERK1/2, HIF-1α, Nanog, Oct4, Sox2, CD44, CD133, and β-actin (dilution 1:2000) overnight at 4 °C. After washing with tris buffered saline with Tween 20 (TBST) three times for a total period of 2 h, secondary antibody against rabbit or mouse (dilution 1:3000) was added, and incubated for 1 h at room temperature. Immunolabeling was detected using an enhanced chemiluminescence (ECL) kit (Bio-Rad Laboratories, Inc.), according to the manufacturer’s instructions.

### 4.9. Tumor Cell-Induced Angiogenesis Assay

A tumor cell-induced chemoinvasion assay was performed using an in vitro co-culture system based on the chemoinvasion assay [48]. The Hep3B cells were seeded in the lower chamber and treated with 10 and 20 μM of α-mangostin, Man-3DG, and Man-6DG for 24 h. The medium in the lower chamber was replaced with fresh medium, and serum-starved HUVECs (8° × 10^4^ cells/well) were added in the upper chamber. The chamber was incubated at 37 °C for 18 h. The HUVECs that invaded the lower chamber were fixed using 70% methanol and stained with hematoxylin and eosin (H&E) at room temperature for 5 min. The total invaded cells were photographed and counted using an optical microscope (Olympus) at a 100× magnification.

To assess the effects of α-mangostin and its glycosides on tumor cell-induced capillary tube formation, a conditioned medium was collected from the Hep3B cells that were treated with 10 and 20 μM of α-mangostin, Man-3DG, and Man-6DG for 24 h, and used as the angiogenic stimuli for the tube formation of HUVECs. Serum-starved HUVECs (2 × 10^3^ cells/well) were seeded on a surface containing Matrigel (10 mg/mL) from an angiogenesis kit (Ibidi GmbH), and treated with the conditioned medium (CM) for 6 h. Tube formation of the HUVECs was photographed and counted using an optical microscope (Olympus) at a 100× magnification.

### 4.10. VEGF Measurement by Enzyme-Linked Immunosorbent Assay (ELISA)

The VEGF concentration in the medium produced from the Hep3B cells that were treated with 10 and 20 μM of α-mangostin, Man-3DG, and Man-6DG was analyzed using a VEGF immunoassay kit (Koma Biotech, Seoul, Korea) following the manufacturer’s instructions. The results were expressed as the concentration of VEGF relative to the total amount of protein from each well.

### 4.11. 3D Cell Culture

The Hep3B cells were cultured in Dulbecco modified Eagle medium/nutrient mixture F-12 (DMEM/F12; Gibco) containing 1× B-27 serum-free supplement (Gibco), 5 μg/mL heparin (Sigma-Aldrich), 2 mM L-glutamine (Gibco), 20 ng/mL epidermal growth factor (EGF; Gibco), 20 ng/mL basic fibroblast growth factor (bFGF; Koma Biotech), and 1% penicillin/streptomycin (Gibco).

### 4.12. CellTiter-Glo^®^ Luminescent Cell Viability Assay

The Hep3B cells (3 × 10^3^ cells/well) were seeded in 96-white-well culture plates. The cells were treated with various concentrations (0–25 μM) of α-mangostin, Man-3DG, and Man-6DG and incubated in serum-free suspended spheroid culture condition for 7 days. Thereafter, 20 μL of substrate solution was added to each well, and the culture plate was shaken for 2 min and incubated in the dark for 8 min. The luminescence was detected using a multimode microplate reader (BioTek, Inc., Winooski, VT, USA).

### 4.13. Tumorsphere Forming Assay

The Hep3B cells (3 × 10^3^ cells/well) were seeded in 96-well culture plates and treated with 3.13 μM of α-mangostin, Man-3DG, and Man-6DG. After incubation in the 3D culture environment for 7 days, the size and number of tumorspheres were confirmed under a 100× optical microscope (Olympus).

### 4.14. Statistical Analysis

All experiments were repeated at least three times. The results were expressed as the mean ± standard deviation (SD). The differences among groups were analyzed using the analysis of variance (ANOVA) with SPSS statistics package (SPSS 9.0; SPSS Inc.). A post-hoc analysis was carried out by Tukey’s test. A *p*-value of <0.05 was considered to indicate a statistically significant difference.

## 5. Conclusions

The present study demonstrates, for the first time, the anticancer effects and underlying molecular mechanisms of two α-mangostin glycosides, including α-mangostin 3-*O-β*-D-2-deoxyglucopyranoside (Man-3DG) and α-mangostin 6-*O-β*-D-2-deoxyglucopyranoside (Man-6DG), against hepatocellular carcinoma (HCC) cells. The results showed that Man-3DG and Man-6DG effectively suppressed the growth, migration, tumor angiogenesis, and cancer stemness of HCC cells in vitro. Furthermore, the α-mangostin glycosides induced G0/G1 cell cycle arrest and mitochondrial caspase-dependent apoptosis in HCC cells. Notably, inhibition of autophagy increased the apoptosis-inducing effect of α-mangostin glycosides against HCC cells. Moreover, the anticancer and antiangiogenic effects of the α-mangostin glycosides were associated with the downregulation of c-Met and HIF-1α activities. Interestingly, Man-6DG showed better therapeutic potential than Man-3DG for HCC cells, implying that the conjugation position of the sugar affects their bioactivities. In addition, Man-6DG exhibited superior anticancer effects to α-mangostin and Man-3DG under a 3D spheroid culture condition of HCC cells. In conclusion, these findings provide support for the potential use of the glycoside analogs of α-mangostin that can have increased water solubility and bioavailability in comparison to α-mangostin in further preclinical and clinical applications for the treatment of HCC.

## Abbreviations

HCC	Hepatocellular carcinoma
Man-3DG	α-Mangostin-3-*O*-*β*-D-2-deoxyglucopyranoside
Man-6DG	α-Mangostin 6-*O*-*β*-D-2-deoxyglucopyranoside
HIF-1α	Hypoxia-inducible factor-1α
VEGF	Vascular endothelial growth factor
HGF	Hepatic growth factor
MAPK	Mitogen activated protein kinase
PI3K	Phosphatidylinositol-3 kinase
AKT	Protein kinase B
STAT	Signal transducer and activator of transcription
FITC	Fluorescein isothiocyanate
PI	Propidium iodide
3-MA	3-Methyladenine
MTT	3-(4,5-dimethylthiazol-2-yl)-2,5-diphenyltetrazolium bromide
ERK	Extracellular signal-regulated kinase
HUVEC	Human umbilical vein endothelial cell
CM	Conditioned medium
AMPK	AMP-activated protein kinase
mTORC1	Mammalian target of rapamycin complex 1
HGFR	Hepatocyte growth factor receptor
PHD	Prolyl-hydroxylase
DMOG	Dimethyloxalylglycine
CSC	Cancer stem cell
Bcl-XL	B-cell lymphoma-extra large
Bax	Bcl-2-associated X protein
Atg5	Autophagy related 5
LC3B	Microtubule-associated proteins 1A/1B light chain 3B
p62	Nucleoporin p62
Oct4	Octamer-binding transcription factor 4
Sox2	Sex-determining region Y-box 2
